# Roburic acid inhibits lung cancer metastasis and triggers autophagy as verified by network pharmacology, molecular docking techniques and experiments

**DOI:** 10.3389/fonc.2024.1449143

**Published:** 2024-10-10

**Authors:** Luyao Wang, Huili Chen, Lili Deng, Mengling Hu, Ziqiang Wang, Kai Zhang, Chaoqun Lian, Xiaojing Wang, Jing Zhang

**Affiliations:** ^1^ Anhui Province Key Laboratory of Clinical and Preclinical Research in Respiratory Disease, Molecular Diagnosis Center, Department of Pulmonary and Critical Care Medicine, First Affiliated Hospital of Bengbu Medical University, Bengbu, China; ^2^ Department of Genetics, School of Life Sciences, Bengbu Medical University, Bengbu, China; ^3^ Research Center of Clinical Laboratory Science, Bengbu Medical University, Bengbu, China; ^4^ Research Center of Clinical Medicine, Bengbu Medical University, Bengbu, China; ^5^ Joint Research Center for Regional Diseases of Institute of Healthcare Management (IHM), The First Affiliated Hospital of Bengbu Medical University, Bengbu, China

**Keywords:** network pharmacology, roburic acid, lung cancer, autophagy, metastasis

## Abstract

**Background:**

Roburic acid (ROB) is a newly discovered tetracyclic triterpene acid extracted from oak galls, which has anti-inflammatory effects, but the mechanism of its anticancer effect is not clear. Our study focuses on exploring the potential mechanism of action of ROB in the treatment of lung cancer using a combination of network pharmacological prediction, molecular docking technique and experimental validation.

**Methods:**

A network pharmacology approach was used to screen the protein targets of ROB and lung cancer, and PPI network analysis and enrichment analysis were performed on the intersecting genes. The tissue and organ distribution of the targets was also evaluated based on the BioGPS database. To ensure the reliability of the network pharmacology prediction results, we proceeded to use molecular docking technique to determine the relationship between drugs and targets. Finally, *in vitro* experiments with cell lines were performed to further reveal the potential mechanism of ROB for the treatment of lung cancer.

**Results:**

A total of 83 potential targets of ROB in lung cancer were collected and further screened by using Cytoscape software, and 7 targets of PTGS2, CYP19A1, PTGS1, AR, CYP17A1, PTGES and SRD5A1 were obtained as hub genes and 7 hub targets had good binding energy with ROB. GO and KEGG analysis showed that ROB treatment of lung cancer mainly involves Arachidonic acid metabolism, Notch signaling pathway, cancer pathway and PPAR signaling pathway. The results of *in vitro* experiments indicated that ROB may inhibit the proliferation and metastasis of lung cancer cells and activate the PPARγ signaling pathway, as well as induce cellular autophagy.

**Conclusions:**

The results of this study comprehensively elucidated the potential targets and molecular mechanisms of ROB for the treatment of lung cancer, providing new ideas for further lung cancer therapy.

## 1 Introduction

Lung cancer remains the leading cause of malignancy-related deaths worldwide, with an estimated 2.09 million new cases and 1.76 million deaths annually (1, 2). Lung cancer is primarily classified as small cell lung cancer (SCLC) or non-small cell lung cancer (NSCLC), with the latter accounting for 80–85% of cases (3, 4). The 5-year overall survival for lung cancer is still low, despite the recent and significant advancements in diagnostic modalities such as tumor biomarkers and treatment techniques such surgery, chemotherapy, radiation, targeted therapy, or immunotherapy (5–7). Therefore, in the realm of lung cancer research, finding novel and efficient therapies is a pressing concern in light of these therapy conundrums.

Traditional Chinese medicine (TCM) has been widely recognized as an alternative or complementary treatment for cancer patients, and an increasing amount of evidence supports its positive effects on the disease (8, 9). One tetracyclic triterpenoid that occurs naturally is called Roburic acid (ROB). ROB was originally isolated from oak galls and subsequently found in plants such as the traditional Chinese medicine *Gentiana macrophylla Pall.* (10, 11). There have been few reports on the biological activity of ROB to date. Y Chen et al. reported tha ROB suppresses NO and IL-6 production via targeting NF-κB and MAPK Pathway in RAW264.7 cells (12). In addition, ROB had beneficial benefits on Alzheimer’s illness (13). Verhoff M, et al. reported that roburic acid failed to significantly inhibit microsomal prostaglandin E2 synthase (mPGES)-1 activity (10). These findings suggest that ROB could be a potential new drug for the treatment of disease. Unfortunately, there aren’t many mechanistic studies on the protective benefits of ROB in cancer. For this reason, the current study uses a network pharmacology method to try to make a crucial first step in this field.

**Figure 1:**
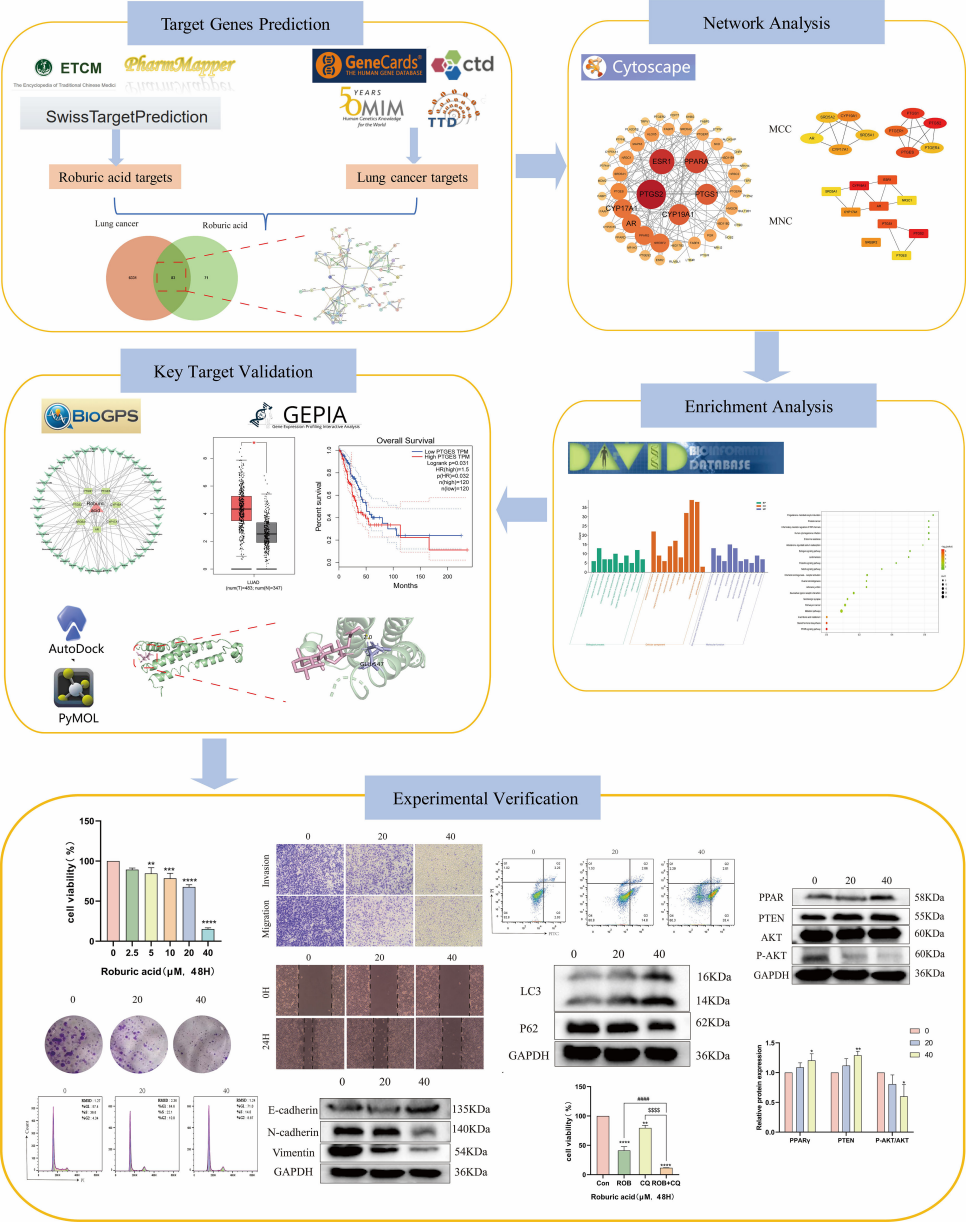
Detailed procedure of ROB treatment for lung cancer.

Network pharmacology, which combines systems biology with information science, is emerging as a new frontier in drug discovery and development (14). It offers a potential research strategy by combining biological, pharmacological and bioinformatics approaches to discover relationships between diseases and active ingredients (15). In the present study, network pharmacology and molecular docking were used to predict potential targets, and signaling pathways of ROB in the treatment of lung cancer. In addition, we evaluated the expression of the hub targets in lung cancer in relation to survival prognosis. Then, cell biological assays were performed on A549 and H1299 cell lines. The detailed process of this study is shown in **Figure 1**.

## 2 Materials and methods

### 2.1 Collection of ROB related target genes

We used three databases, ETCM (http://www.tcmip.cn/ETCM/index.php/Home/), PharmMapper (http://bionet.ncpsb.org.cn/batman-tcm/index.php) and Swiss Target Prediction (https://labworm.com/tool/swisstargetprediction), and used “Roburic acid” as the query target. For the Swiss Target Prediction database, targets with probability value > 0 were selected. For the PharmMapper database, the targets with Norm Fit > 0.8 were selected and converted to gene names using the UniProt database (https://www.uniprot.org/). The targets from these three databases were then merged and removed duplicates, and the remaining targets were the ROS targets we collected.

### 2.2 Identification of predicted targets of lung cancer

Target genes for lung cancer were obtained using GeneCards (https://www.genecards.org/), Online Mendelian Inheritance in Man (OMIM, https://www.omim.org/), Therapeutic Target database (TTD, http://ctdbase.org/) and The Comparative Toxicogenomics database (CTD, https://ctdbase.org/) with lung cancer as the keyword. Lung cancer genes were obtained by screening based on a relevance score of ≥5 in the GeneCards database for subsequent analysis. Duplicate genes from the four disease database targets were eliminated to obtain lung cancer-related target genes.

### 2.3 Construction of protein-protein interaction network

To collect overlapping targets of ROB and lung cancer, we imported the targets of both into the online software jevnn (https://www.bioinformatics.com.cn/static/others/jvenn). The targets in the intersection set were imported into the STRING database (https://string-db.org/) to obtain information on protein interaction networks (16). In this database, the screening condition for organisms is set to “Homo sapiens” and the minimum required interaction score is “high confidence (0.7)”. The PPI data was then imported into Cytoscape 3.9.0 software tomap the PPI network. Continuing in the Cytoscape software, the parameters of each node in the network graph were calculated, where the degree of freedom was used as topological indices, which are commonly used as indicators to describe the importance of the network nodes. Finally, the hub genes were further mined using CytoHubba and MCODE plugins.

### 2.4 GO and KEGG enrichment analyses

We imported ROB-lung cancer targets into the DAVID (https://david.ncifcrf.gov/) and KOBAS (http://kobas.cbi.pku.edu.cn/kobas3) databases for the Gene Ontology (GO) annotation and Kyoto Encyclopedia of Genes and Genomes (KEGG) enrichment analysis. GO analysis focuses on biological processes (BP), cellular components (CC) and molecular functions (MF). Finally, the data were uploaded to a bioinformatics (http://www.bioinformatics.com.cn/) platform for visualization and analysis. To illustrate the relationship between drugs, targets, and pathways, we also built compound-target-pathway networks and evaluated them using Cytoscape software.

### 2.5 Construction of organ-targets network

The expression levels of mRNAs for each pharmacological target in organs and tissues were obtained using the BioGPS database (17). The findings demonstrated that U133 affinity microarrays were examined in various normal human tissues (18). ROB-lung cancer targets were imported into this database, probes with high correlation were selected and visualized using Cytoscape software.

### 2.6 Validation of hub targets

The hub targets were validated in cancer tissues using the Gene Expression Profiling Interactive Analysis (GEPIA, http://gepia.cancer-pku.cn/index.html) database, GEPIA database and further analysis of hub gene expression and survival prognosis in lung cancer and normal lung tissue.

### 2.7 Molecular docking

Download the structure (.sdf format) of ROB in the PubChem database (https://pubchem.ncbi.nlm.nih.gov/) and converted to mol2 format using OpenBabel software (https://openbabel.org). The structures of the screened target proteins (.pdb format) were obtained from the PDB database (https://www.rcsb.org/). We first removed water molecules and ligands from the key protein structures in Pymol software, then we imported them into AutoDock Tools software for docking and set up docking boxes to obtain the minimum binding energy. Finally, the Pymol software was used for visualization and analysis.

### 2.8 Cell culture

Lung cancer cells A549 and H1299 cells were cultured in DMEM and RPMI-1640 medium (Gibco, ThermoFisher Scientific, United States) containing 10% fetal bovine serum (FBS) and 1% penicillin-streptomycin in a 5% CO2 humidified incubator at 37°C. ROB purchased from MCE (Hefei, China) and dissolved in DMSO.

### 2.9 Cell proliferation assays

In this study, the Cell Counting Kit-8 (CCK-8) assay was used to determine cell proliferation. A549 and H1299 cells were inoculated into 96-well plates with 5 × 10^3^ cells per well and incubated overnight in the incubator. The next day, the cells were treated with ROB (0, 2.5, 5, 10, 20 and 40 μM) for 48 h, followed by the addition of 10 μl of CCK8 solution (Bimake, USA) to each well and then incubated for 2 h. Finally, the OD value was measured at 450nm using a microplate reader (BioTek, cytation3, USA). For colony formation assay, 1× 10^3^ cells were seeded into 6-well plates and cultured overnight, with fluid changes every two days. 15 days after ROB treatment for 48 h. Subsequently, the cells were fixed in 4% paraformaldehyde for 10 min, washed twice, and then stained with 1% crystal violet staining solution, and finally the number of colonies was counted using ImageJ software.

### 2.10 Migration and invasion assays

Transwell assay was used to determine cell invasion and migration. After 24 h of ROB action, A549 and H1299 cells (4 × 10^4^) were suspended in serum-free medium, and the invasion experiments and the suspension (200 μl/well) was added to the upper chamber pre-coated with matrix gel, whereas migration experiments did not require pre-coating with matrix gel; 800 μl of serum-containing medium (10% FBS) was added to the lower chamber. After 48 h of incubation, non-migrating cells on the upper surface of the membrane were removed with a cotton swab, and cells penetrating the lower surface of the membrane were stained with crystal violet. Cell counting was performed using ImageJ software. Subsequently, a wound healing assay was performed to detect cell migration ability. A549 and H1299 cells were inoculated into a 12-well plate, cultivation using FBS-free basal medium and when the cells reached 90 or more, a straight line was scraped with the tip of a 200 μl pipette and ROB was added to treat the cells. At 0 h and 24 h, cell images were captured and the wound closure area was measured.

### 2.11 Apoptosis and cell cycle assays

Apoptosis was detected using the FITC Annexin V Apoptosis Detection kit (BD, USA). A549 and H1299 cells were treated with different concentrations of ROB for 48 h. Cells were washed twice with pre-cooled PBS, resuspended in 1 × binding solution, and added 5 μl of FITC Annexin V and 5 μl PI. The cells were gently mixed and incubated in the dark for 20 min at room temperature. 400 μl 1 × binding solution was added to each tube and measured by flow cytometry (BD Biosciences, USA) within 1 h. A cell cycle staining kit (Beyotime, Shanghai, China) was used to analyze the cell cycle. ROB-treated cells were collected, washed once with pre-cooled PBS, fixed with pre-cooled 70% ethanol at 4°C overnight, and then stained with PI/RNase mixture for 30 min at 37°C in the dark, and finally detected by flow cytometry.

### 2.12 Western blot assays

After ROB treatment for 48 h, the cells were lysed with RIPA lysate (Servicebio, China) on ice and the supernatant was collected, the extracted proteins were added to the appropriate amount of upwelling buffer, and the denaturation was accomplished by boiling for 5-10 min in a 95°C water bath. Proteins were subsequently separated by sodium dodecyl sulfate-polyacrylamide gel electrophoresis (SDS-PAGE). Isolated proteins were transferred to a PVDF membranes (Immobilon-P, Carlsbad, Ireland), closed with 5% skimmed milk for 2 h, and incubated with primary antibody at 4°C overnight. On the following day, the membrane was washed 3 times with 1×TBST for 10 min each time, and then the membrane was put into the diluted secondary antibody and shaken for 2 h at room temperature on a shaker. Then, the enhanced ECL chemiluminescence detection kit as used to visualize protein signals. Finally, the strips were measured using ImageJ software. Primary antibodies used: E-Cadherin (GB111273, Servicebio), N-Cadherin (GB11082, Servicebio), Vimentin (GB111308, Servicebio), LC3 (12741S, Cell Signaling Technology), P62 (A5180,Bimake), PPARγ (16643, Proteintech), PTEN (60300, Proteintech), Phospho-AKT (4058S, Cell Signaling Technology), AKT (A5023, Selleckchem), GAPDH (GB11002, Servicebio).

### 2.13 Statistical analysis

The data are shown as were displayed as the mean ± standard deviation (SD). Statistical analyses were performed using GraphPad Prism 8.0 software, with one-way analysis of variance (ANOVA) for comparisons between multiple groups and t-test for comparisons between two groups. p<0.05 was considered statistically different and at least three trials were performed for each experiment.

## 3 Results

### 3.1 Acquisition of potential targets for ROB action in lung cancer

**Figure 2:**
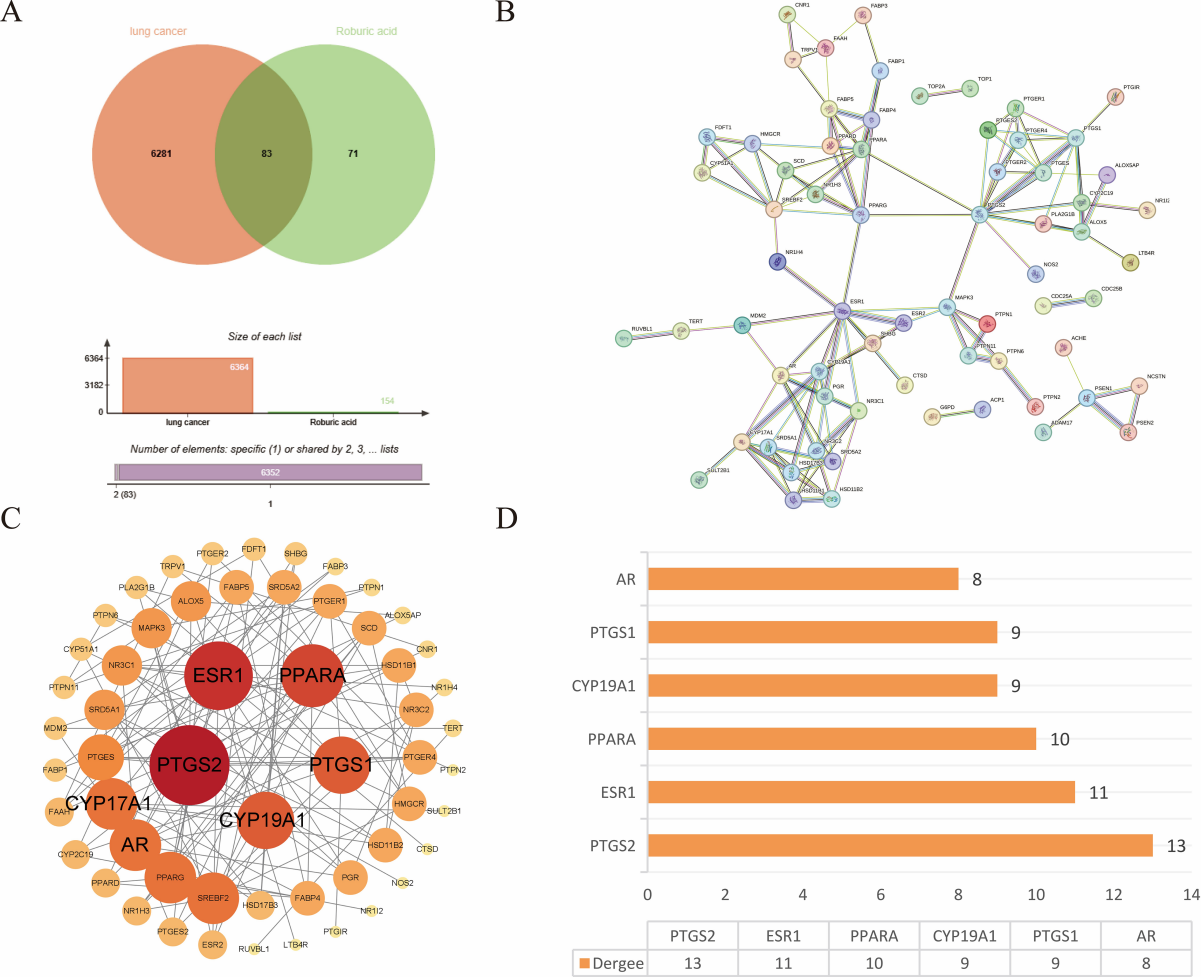
Target acquisition and construction of PPI network for ROB action in lung cancer. **(A)** Venn diagram of intersecting genes for ROB and lung cancer targets. **(B)** *STRING* database to obtain PPI network. **(C)** 83 intersecting genes were visualized by Cytoscape, and the size and color of the nodes represent the magnitude of the Degree. **(D)** Histogram of the first six genes with higher Degree values.

The targets of ROB were obtained from three databases, ETCM, PharmMapper and Swiss Target Prediction databases, where ETCM database obtained 2 targets (**Supplementary Table S1**), PharmMapper database obtained 62 targets (**Supplementary Table S2**) and Swiss Target Prediction database obtained 100 targets (**Supplementary Table S3**). The three databases were combined and de-emphasized to obtain 154 targets for ROB. From the GeneCards database, screening based on relevance score ≥5 (**Supplementary Table S4**) resulted in 6003 lung cancer-related genes. In addition, 504 targets for lung cancer were obtained in the OMIM database (**Supplementary Table S5**). Then, 161 and 91 potential targets for lung cancer were obtained in the TTD and CTD databases (**Supplementary Table S6**). Finally, 6364 targets for lung cancer were obtained. At last, the final 83 overlapping targets between ROB and lung cancer as possible therapeutic targets (**Figure 2A**, **Supplementary Table S7**).

### 3.2 Construction of PPI network and screening of hub genes

**Figure 3:**
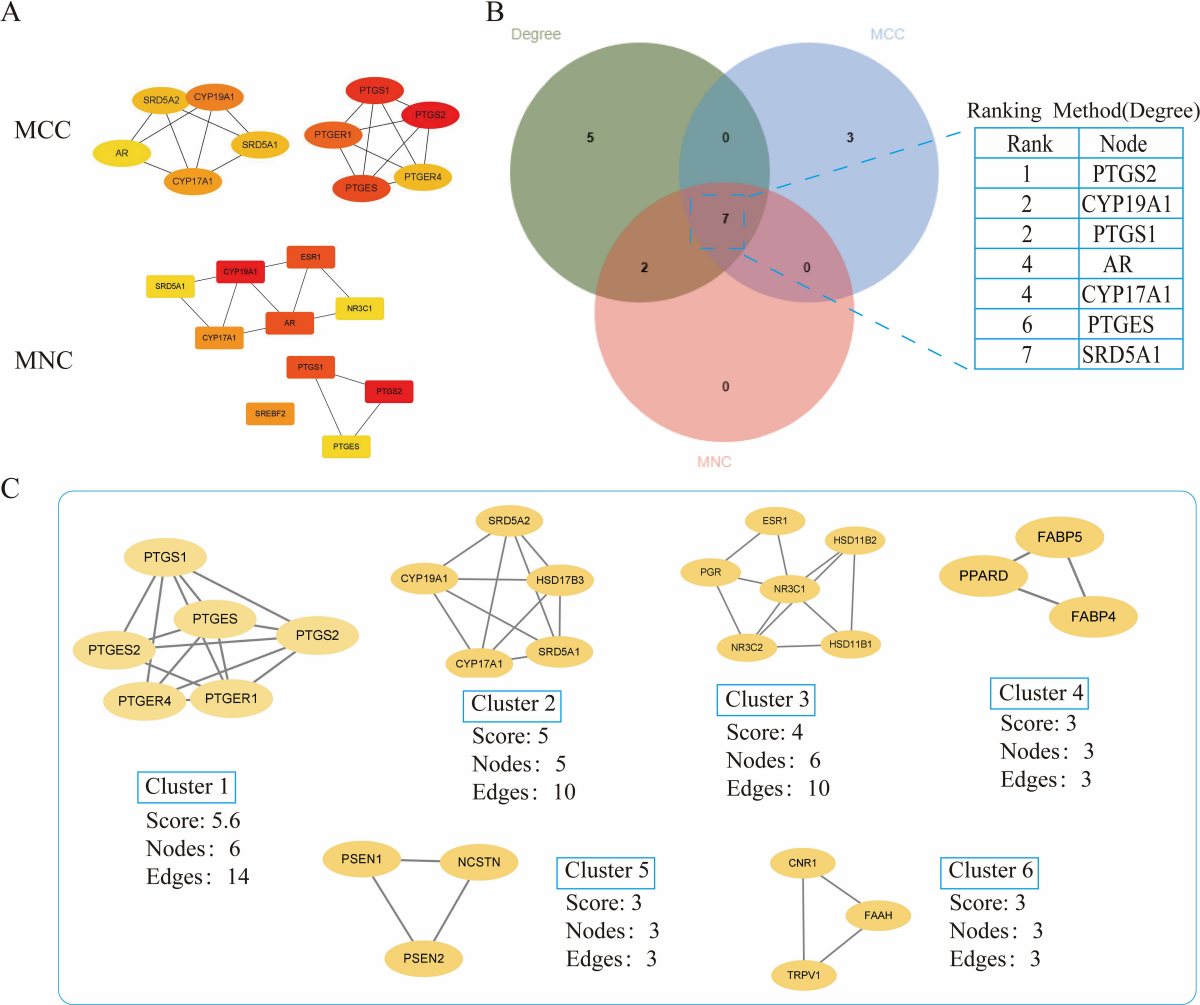
Further screening of hub genes by Cytoscape. **(A)** Topology analysis based on CytoHubba, MCC and MNC screened for 10 hub genes. Yellow to red color indicates the degree value of the target in the PPI network. **(B)** Venn diagram of Degree, MCC, and MNC screened 7 hub genes **(C)** Clustering analysis of hub genes by MCODE plugin. The highest gene scores were found in Cluster 1.

The crossover genes were submitted to the STRING database and a PPI analysis was performed (**Figure 2B**). A total of 83 nodes and 131 edges were obtained with an average node degree of 3.16. The interacting targets were then introduced into Cytoscape software. Based on the Degree value, the top 6 targets, including PTGS2, ESR1, PPARA, CYP19A1, PTGS1, and AR were used as the hub targets (**Figure 2C**), and bar graphs were drawn (**Figure 2D**). In addition, hub genes were further screened using the maximal clique centrality (MCC) algorithm and the maximum neighborhood component (MNC) algorithm in Cytoscape’s plug-in Cytohubba (**Figure 3A**). The hub targets of ROB action in lung cancer were further analyzed by combining 14 targets from Degree≥6, 10 targets each from MCC and MNC algorithms, and finally 7 common targets such as PTGS2, CYP19A1, PTGS1, AR, CYP17A1, PTGES and SRD5A1 were obtained from the final screening (**Figure 3B**). The detailed information is shown in **Table 1**. Furthermore, clustering analysis was performed by the MCODE plugin of Cytoscape software to generate highly connected sub-networks and assign targets to six groups (**Figure 3C**). The highest score was cluster 1 containing 6 nodes and 14 edges.

**Table 1:** Hub targets with key roles in ROB therapy for lung cancer.

Targets	Degree	Betweenness Centrality	Closeness Centrality
PTGS2	13	0.456424	0.413534
CYP19A1	9	0.112368	0.325444
PTGS1	9	0.043490	0.314286
AR	8	0.065336	0.327381
CYP17A1	8	0.042677	0.264423
PTGES	7	0.019024	0.307263
SRD5A1	6	0.003236	0.258216

### 3.3 GO enrichment analysis

**Figure 4:**
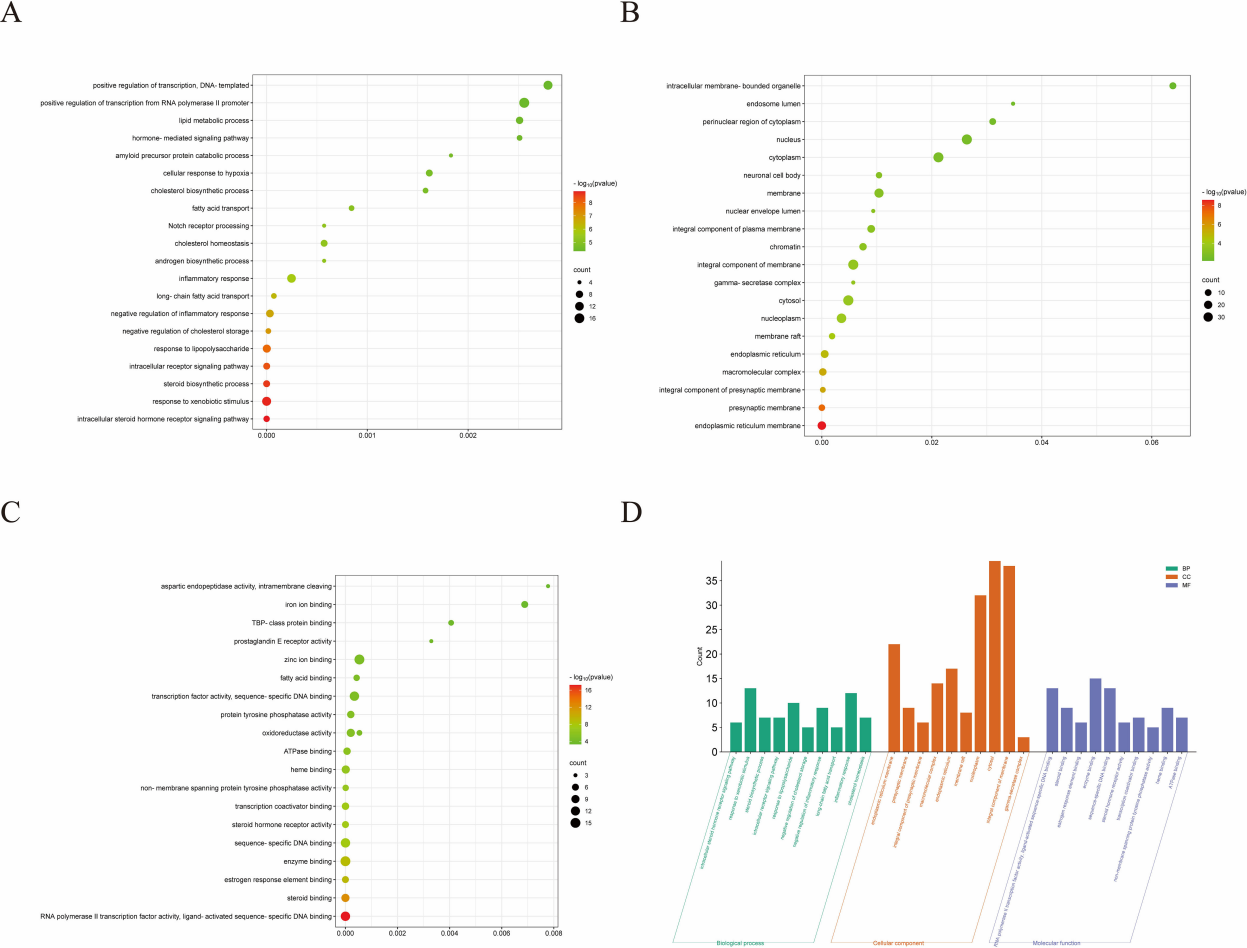
GO enrichment analysis of ROB treatment for lung cancer. **(A–C)** Bubble charts for the first 20 items of Biological Processes (BP), Cellular Components (CC), and Molecular Functions (MF). **(D)** The bars of the 10 significantly enriched GO pathways are shown in green, orange, and purple to represent the enrichment analysis of BP, CC and MF.

To further investigate the molecular mechanism of ROB in the treatment of lung cancer, we subjected 83 intersecting targets to GO and KEGG enrichment analysis. The results of the GO analysis were obtained using the DAVID database (**Supplementary Table S8**), and the top 20 items enriched in BP (**Figure 4A**), CC (**Figure 4B**), and MF (**Figure 4C**) are represented as bubble plots. BP were mainly associated with intracellular receptor signaling pathway, steroid biosynthetic process, response to xenobiotic stimulus and intracellular steroid hormone receptor signaling pathway, CC with macromolecular complex, integral component of presynaptic membrane, presynaptic membrane, endoplasmic reticulum membrane etc., and MF with steroid binding,enzyme binding, sequence- specific DNA binding, estrogen response element binding etc. . In addition, GO enrichment analysis was also used for enrichment scores, which are shown as bar graphs (**Figure 4D**).

### 3.4 KEGG enrichment analysis

**Figure 5:**
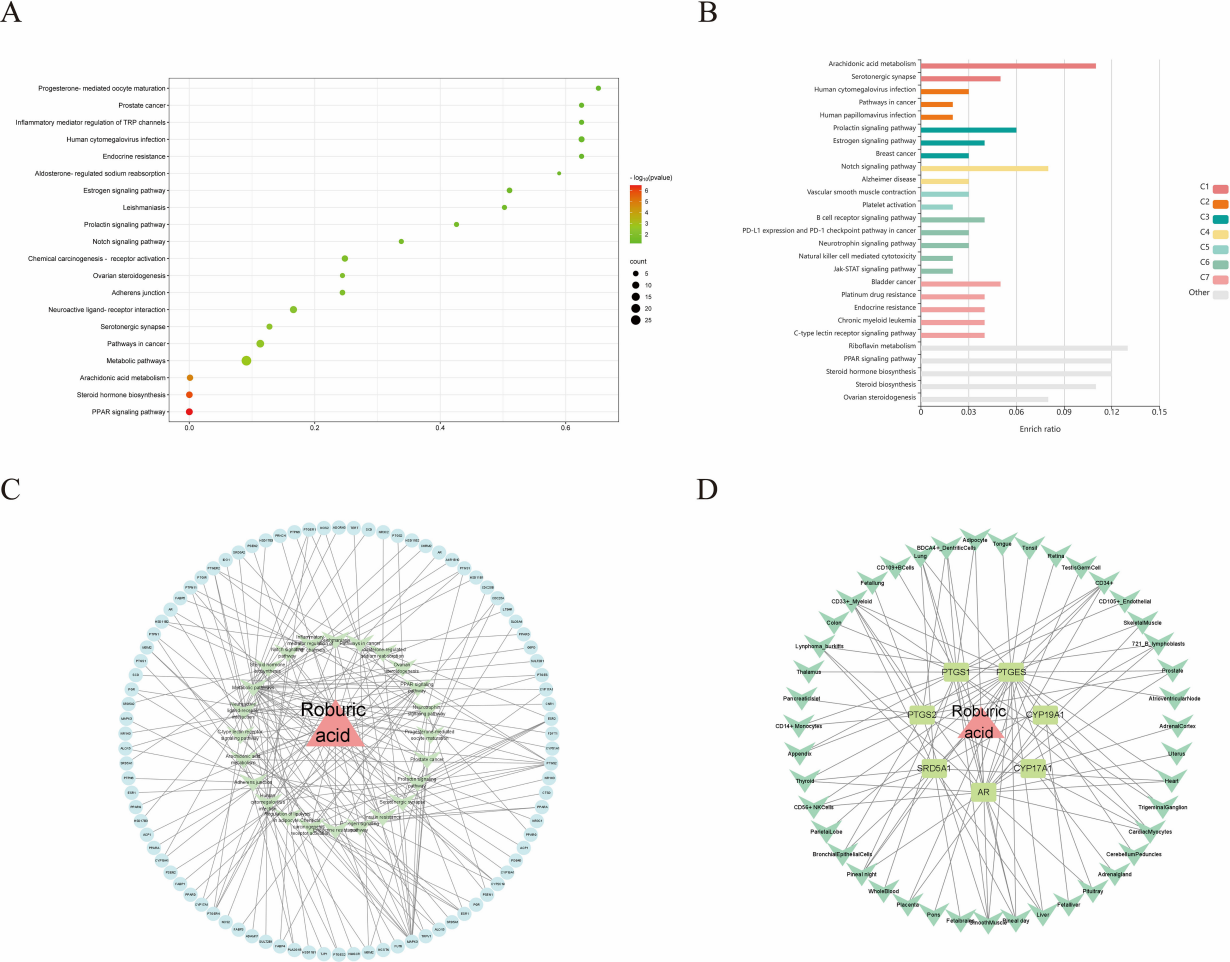
ROB acts on KEGG enrichment, pathway networks and tissue target networks in lung cancer. **(A)** Bubble plots of the top 20 pathways to obtain KEGG enrichment results for ROB action in lung cancer at the DAVID database. **(B)** Bar graph of KEGG enrichment results of ROB action in lung cancer obtained from KOBAS database. **(C)** Network diagram of drug-target-pathway of ROB for the treatment of lung cancer. The pink triangle represents the drug ROB, the light blue original shape represents the target and the light green V-shape represents the pathway. **(D)** Drug-target-tissue network of 7 hub targets. Pink triangles represent drug ROB, light green rectangles represent targets, and green V-shapes represent tissues.

For KEGG enrichment analysis, we used two databases, DAVID and KOBAS, to ensure the accuracy of the results. In DAVID database, there were 24 KEGG enrichment items in total, the enrichment results mainly include PPAR signaling pathway, Notch signaling pathway, Pathways in cancer and Steroid hormone biosynthesis etc (**Figure 5A**, **Supplementary Table S9**). In KOBAS database mainly include Arachidonic acid metabolism, Prolactin signaling pathway, Jak-STAT signaling pathway, Notch signaling pathway, B cell receptor signaling pathway and PPAR signaling pathway etc (**Figure 5B**). Both the Notch signaling pathway and PPAR signaling pathway are present in both databases, but the PPAR signaling pathway is in the DAVID database with a more meaningful p-value, assuming that ROB acts through the PPAR signaling pathway in lung cancer cells. In order to elucidate the mechanism of ROB in treating lung cancer through multiple pathways and targets, the KEGG enrichment results were imported into Cytoscape software to visualize the ROB-targets-pathways network (**Figure 5C**), and these signaling pathways and targets may be the key mechanisms of ROB in treating lung cancer.

### 3.5 Construction of organ-target network

Evaluating mRNA levels of 7 hub targets based on the BioGPS database (**Figure 5D**). Based on the BioGPS database to assess the mRNA levels of 7 hub targets, the hub targets had elevated mRNA levels in lung cancer-related organs, including lung, SmoothMuscle, Cd34+ cells, cd33+ cells and Cd56+ cells. Most of the targets showed significantly high expression in multiple tissues and organs of lung cancer at the same time, indicating that these organs were closely related to the targets of lung cancer. In addition, these organs were closely related to immunity, suggesting that the therapeutic effects of ROB on lung cancer involve various tissues throughout the body.

### 3.6 Expression of hub targets and prognosis

**Figure 6:**
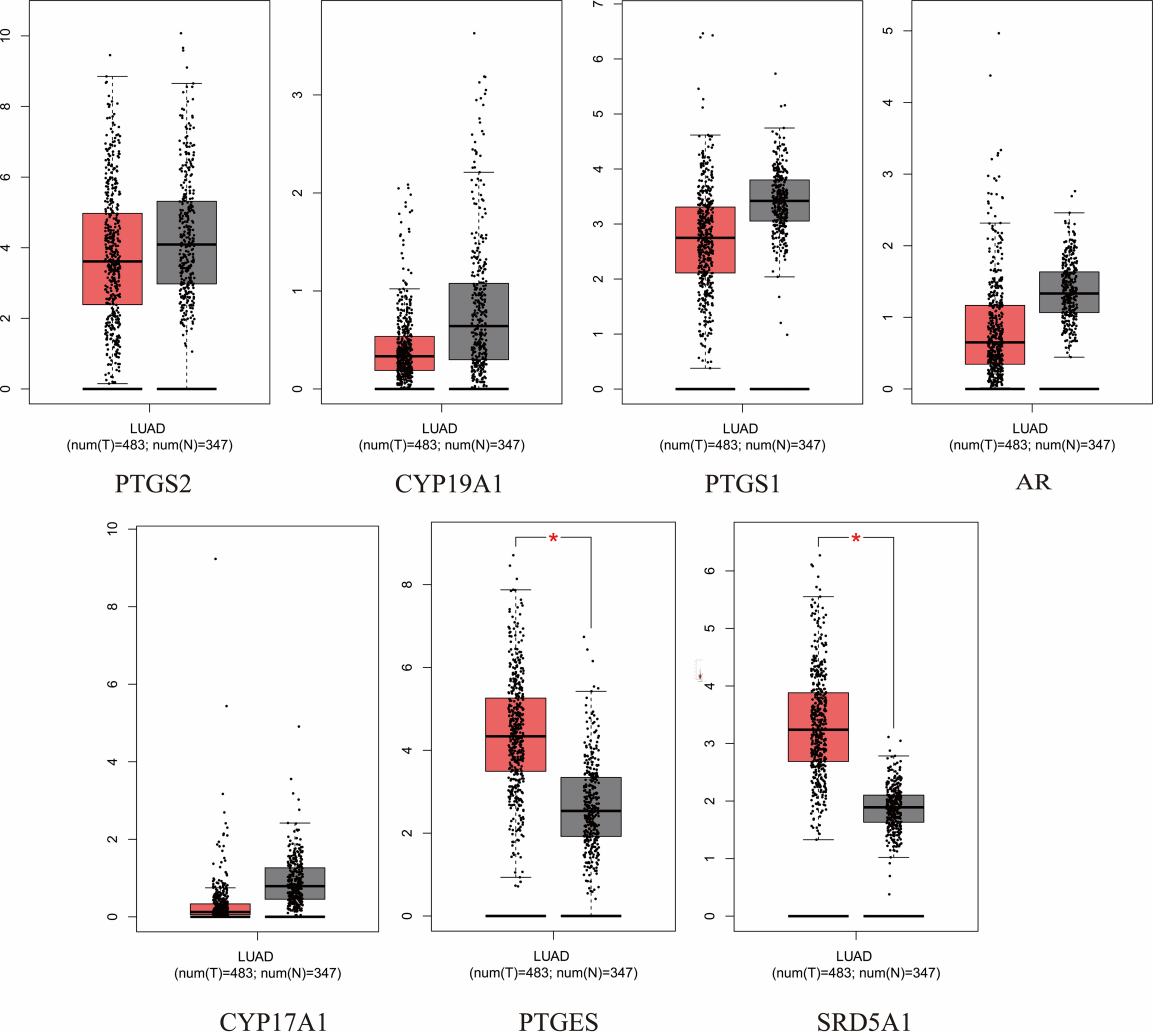
Expression of 7 hub genes in the GEPIA database in lung cancer versus normal lung tissue. *P<0.05 vs. normal tissue.

**Figure 7:**
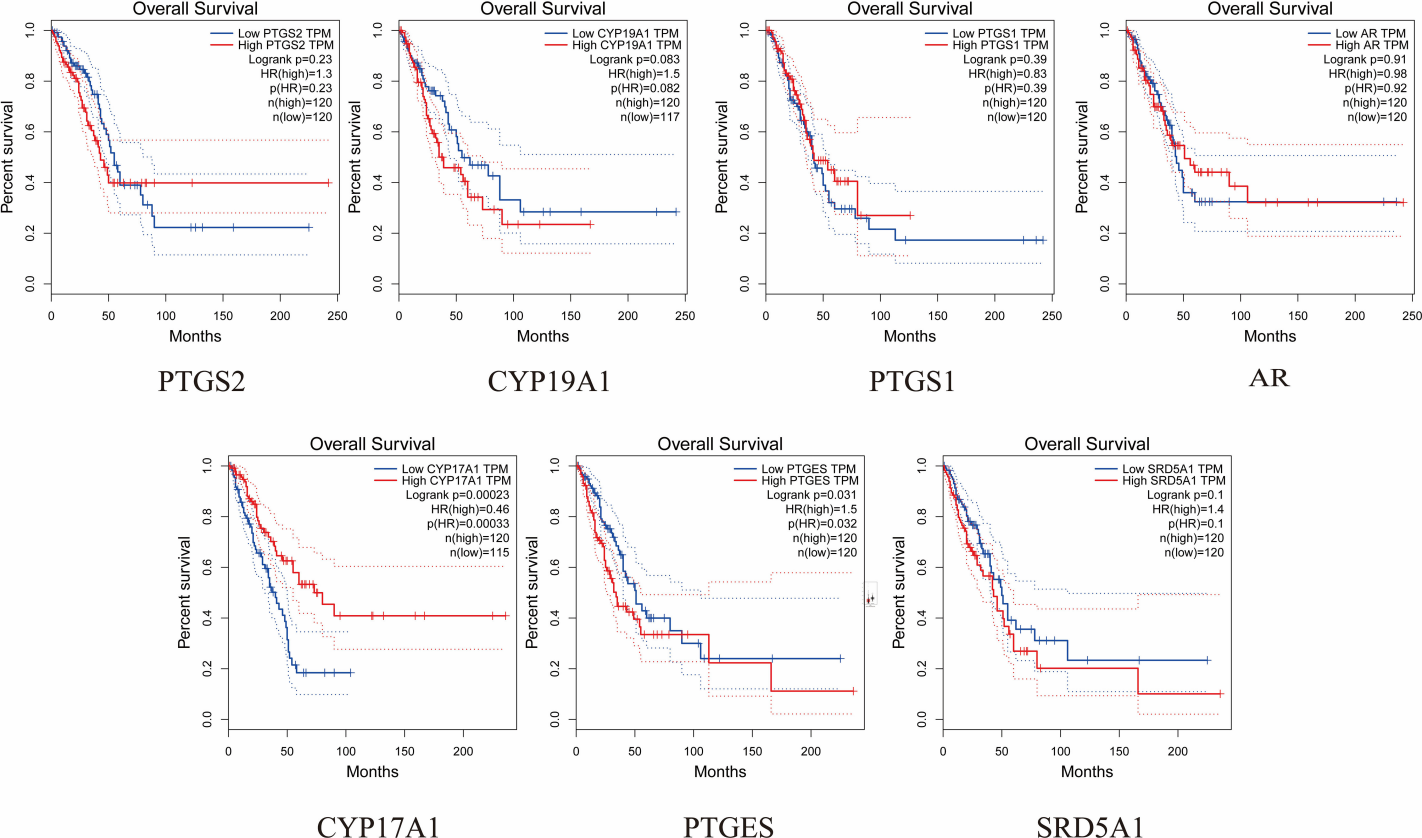
Overall survival of the 7 hub genes in the GEPIA database.

The expression and prognosis of hub genes in lung adenocarcinoma (LUAD) samples were studied using the GEPIA database. The results showed that among the 7 hub genes, PTGES and SRD5A1 were highly expressed in LUAD, which was significant (P<0.05) (**Figure 6**). To further elucidate the prognostic value of these hub genes, we analyzed the Overall survival (OS) of LUAD patients. Overall survival results showed that high expression of PTGES was significantly associated with low survival, while high expression of CYP17A1 was associated with high survival (P<0.05) (**Figure 7**). In conclusion, only overexpression of PTGES resulted in poorer prognosis. Therefore, ROB targeting PTGES may be meaningful targets for the treatment of lung cancer.

### 3.7 Molecular docking validation

**Figure 8:**
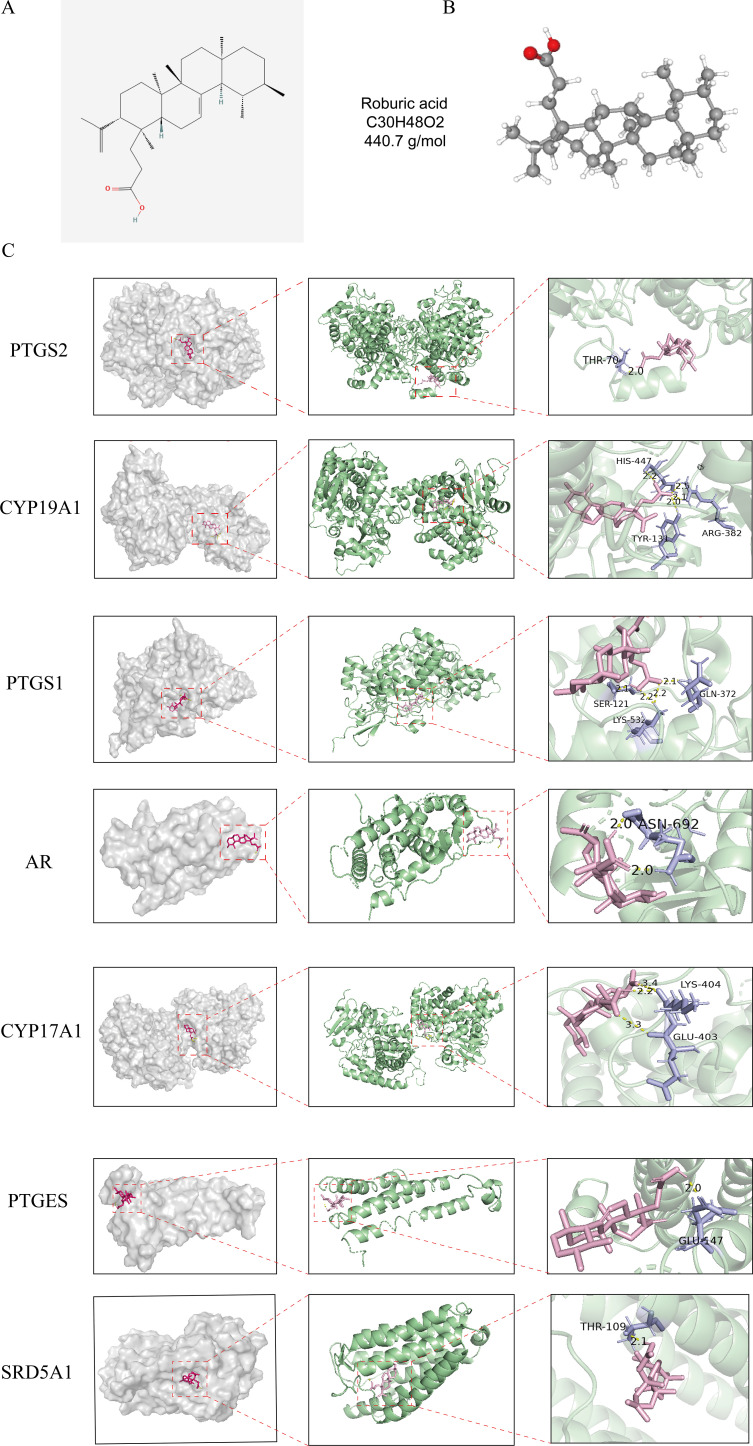
Structure of ROB and its 3D binding and hydrogen bonding diagram with the hub targets. **(A, B)** 2D and 3D structural diagrams of ROB. **(C)** Molecular docking map of ROB with 7 hub targets. The pink image represents the ROB, the purple rods are the amino acid residues where the ROB binds to the core target, and the binding sites are connected by yellow hydrogen bonds.

2D and 3D structure maps of ROB were obtained from the PubChem database (**Figures 8A, B**). Based on the above analysis, the core component ROB was molecularly docked to the core target. Based on the above analysis, ROB was molecularly docked with all 7 core targets (**Figure 8C**). Normally, we consider that the binding energy of docking is < -5 kcal/mol, and there is a good binding ability between the two. Using AutoDock Tools software, the docking results showed good binding between ROB and all 7 core targets (**Table 2**). It suggests that these proteins may be potential binding targets for ROB.

**Table 2:** Binding energy of ROB to 7 hub targets.

Target	PDB ID	Bonding energy (kcal/mol)
PTGS2	3NT1	-6.27
CYP19A1	6UZE	-7.94
PTGS1	6Y3C	-8.13
AR	4LBS	-6.55
CYP17A1	6WR1	-6.48
PTGES	4YL0	-9.0
SRD5A1	7C83	-8.92

### 3.8 ROB inhibits lung cancer cells proliferation

**Figure 9:**
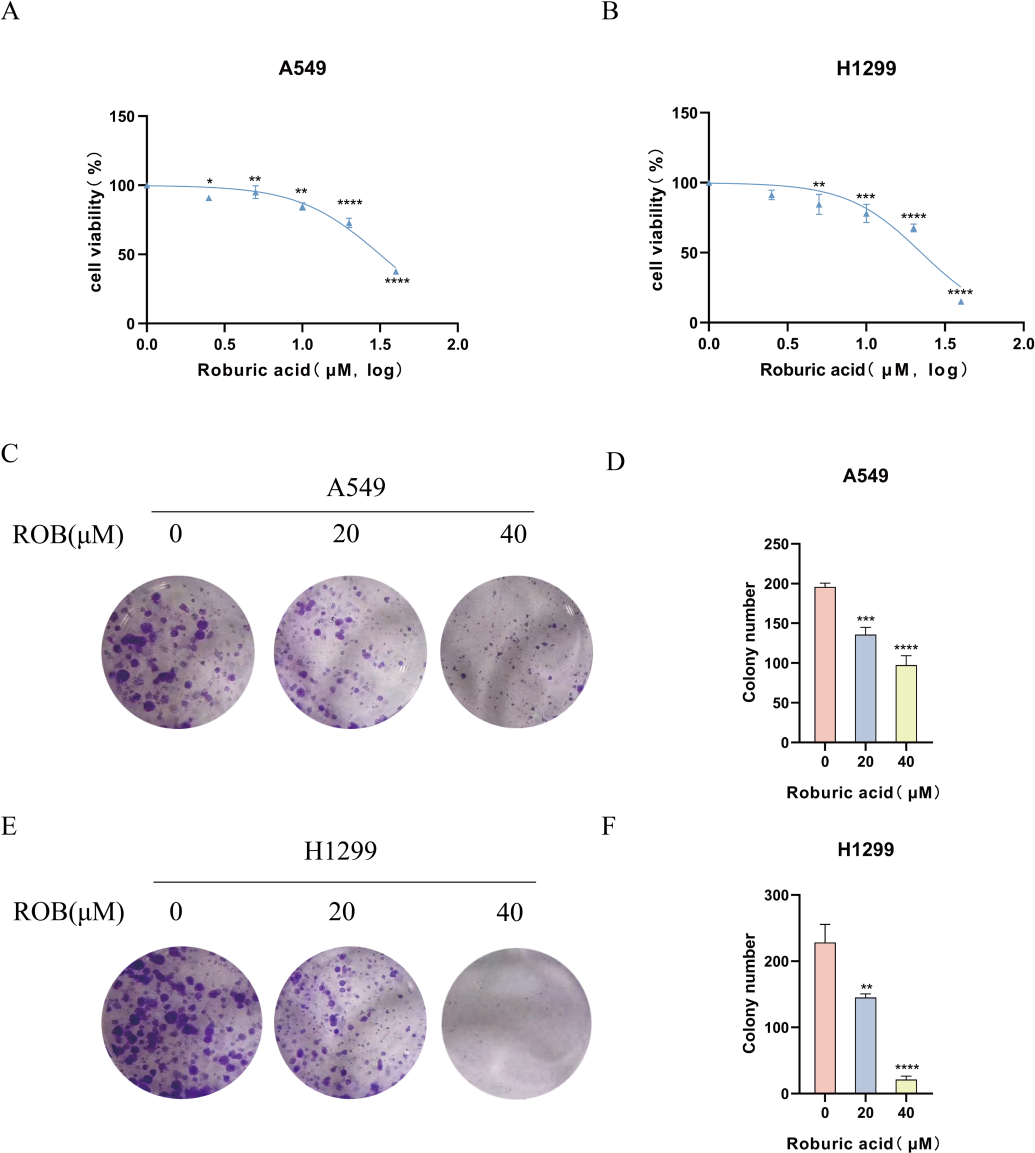
ROB inhibits lung cancer cell proliferation. **(A, B)** Different concentrations of ROB were applied to lung cancer cells for 48 h. Cell viability was determined by CCK8 assay. *p < 0.05, **p < 0.01, ***p < 0.001 and ****p < 0.0001 vs. control group. **(C-F)** Lung cancer cells were treated with ROB for 48 h for colony formation assay. **p < 0.01, ***p < 0.001 and ****p < 0.0001 vs. control group.

To determine the effect of ROB on the proliferative capacity of lung cancer cells, we performed a CCK-8 assay and a colony formation assay. The results of the CCK-8 assay showed that when the ROB concentration was gradually increased from 0 to 40 μM, the cell survival rate of A549 and H1299 cells declined with the increase of the dose (**Figures 9A, B**). ROB exhibited an effective inhibitory effect on lung cancer cells at high concentrations. Then, 0, 20 and 40 μM of ROB were selected for subsequent experiments. In addition, the results of colony formation experiments showed that the number of colonies decreased significantly with increasing ROB concentration compared to the control group (**Figures 9C–F**). These data suggest that ROB inhibits cell viability and colony formation in lung cancer cells.

### 3.9 ROB suppresses invasion and migration of lung cancer cells

**Figure 10:**
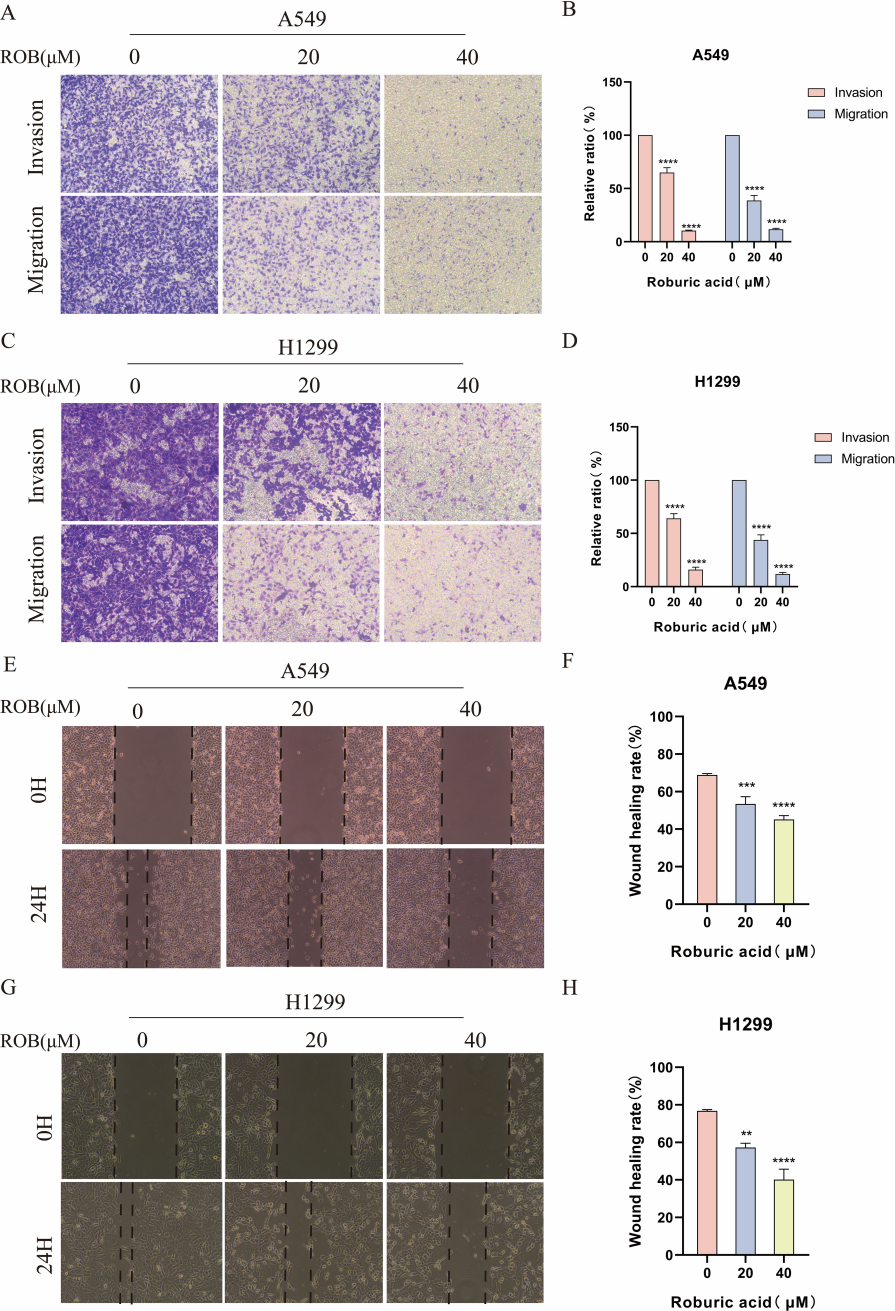
ROB inhibits lung cancer cell invasion and migration. **(A–D)** Lung cancer cells were treated with different doses of ROB for 24 h. Transwell assay was used to detect the migration and invasion ability of lung cancer cells. ****p < 0.0001 vs. control group. **(E–H)** A wound healing test was performed to measure the migration ability of lung cancer cells after 24 h of ROB treatment. **p < 0.01, ***p < 0.001 and ****p < 0.0001 vs. control group.

To determine the effect of ROB on the invasive and migratory capacity of lung cancer cells, we performed transwell assays and wound healing assays. In transwell experiments, the higher the ROB concentration, the fewer cells crossed the luminal membrane (**Figures 10A–D**). It suggests that ROB has an effective inhibitory effect on the invasion and migration of lung cancer cells. A similar phenomenon was observed in wound healing experiments, where 24 h healing was slower in the high ROB concentration group compared to the control group (**Figures 10E–H**). Furthermore, ROB changed in lung cancer cells migration and invasion were dose-dependent.

### 3.10 ROB inhibits EMT in lung cancer cells

**Figure 11:**
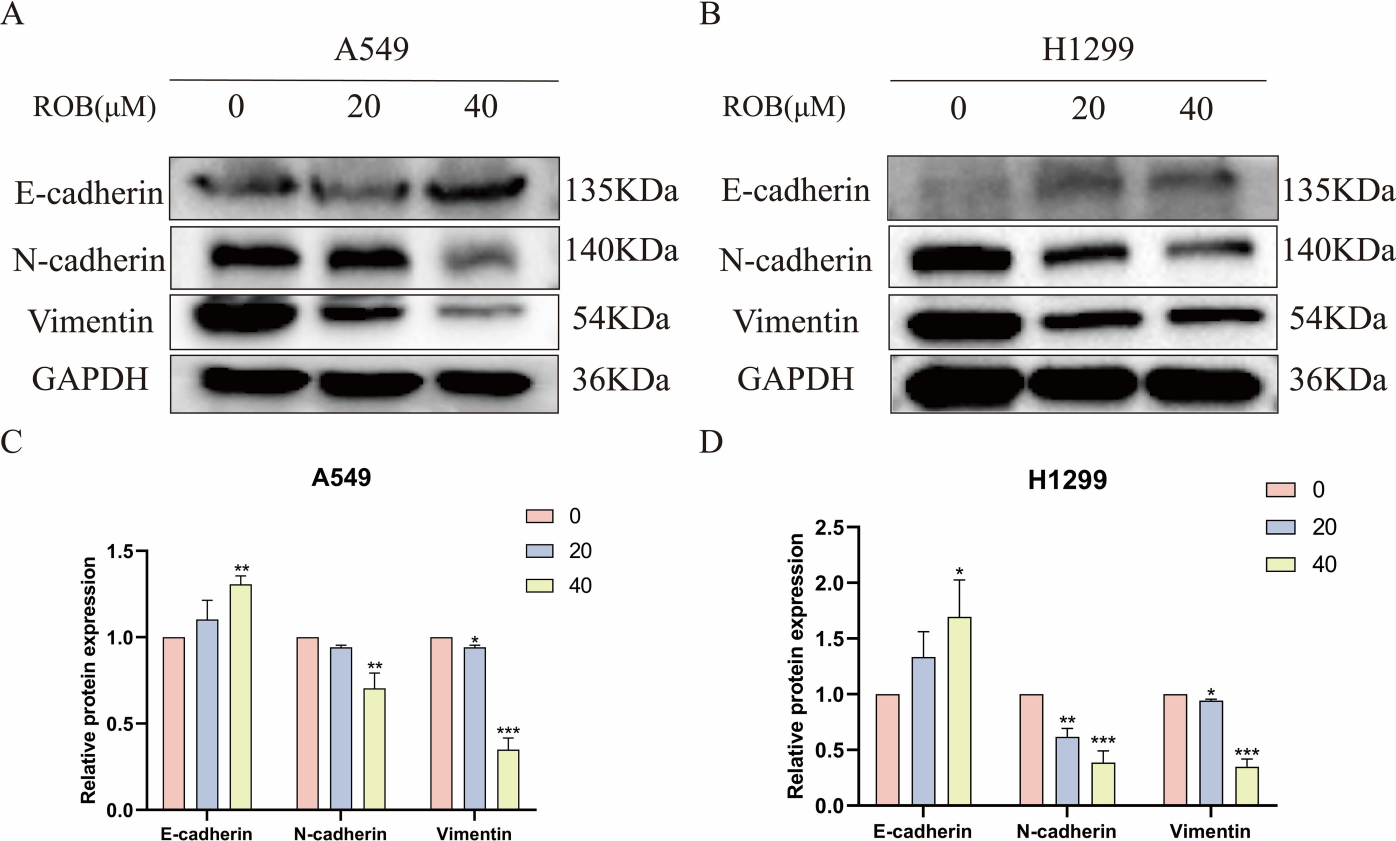
ROB inhibits lung cancer cell EMT. **(A–D)** The expression levels of E-cadherin, N-cadherin and Vimentin were detected by western blotting after 48 h of ROB action on lung cancer cells. *p < 0.05, **p < 0.01 and ***p < 0.001 vs. control group.

EMT plays an important role in the dynamic physiological process of cellular metastasis. We detected the expression of EMT markers, including E-cadherin, N-cadherin, and Vimentin. Western blotting assay showed that 20 μM and 40 μM ROB acted on lung cancer cells and increased the expression of E-cadherin and decreased the expression of N-cadherin and Vimentin protein compared with the control group. In summary, ROB may inhibit EMT in lung cancer (**Figure 11**).

### 3.11 ROB induces cell cycle G1 phase block and apoptosis in lung cancer cells

**Figure 12:**
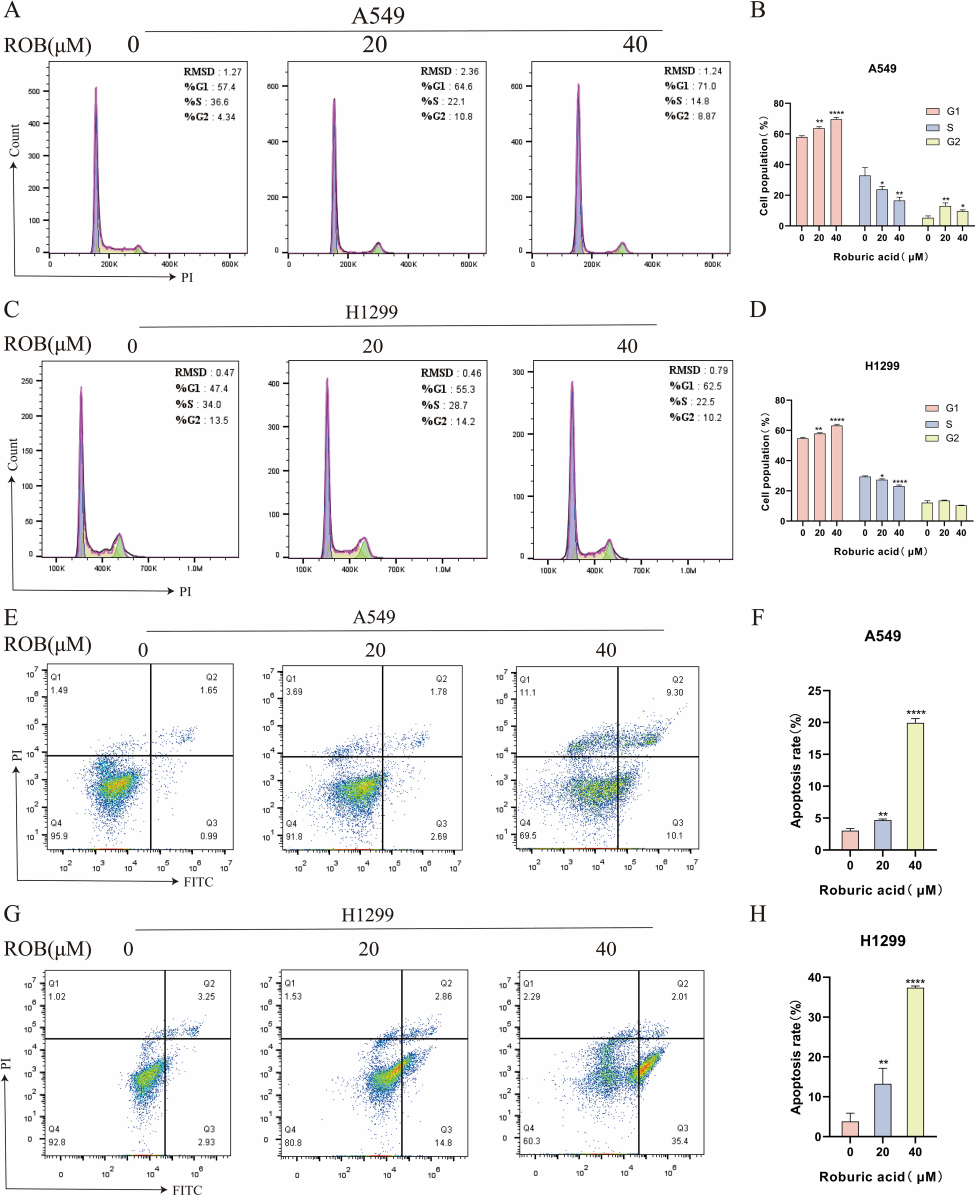
ROB causes cell cycle arrest and apoptosis in lung cancer cells. **(A–D)** The cell cycle of lung cancer cells was detected by PI staining after 48 h of ROB action on lung cancer cells. State the percentage of cells in each cell cycle phase. *p < 0.05, **p < 0.01 and ****p < 0.0001 vs. control group. **(E–H)** After 48 h of ROB action on lung cancer cells, apoptotic cells were detected by Annexin V/PI staining, and the apoptotic rate was determined by flow cytometry. **p < 0.01 and ****p < 0.0001 vs. control group.

Flow cytometry was used to detect the effect of ROB on the cell cycle of A549 and H1299 cells, and the results showed that the proportion of cells in G1 phase gradually increased with the increase of drug concentration, while the percentage of cells in S phase was lower, and there was no significant difference in the percentage of cells in G2 phase (**Figures 12A–D**). This suggests that ROB is able to inhibit the growth of lung cancer cells by blocking cellular DNA synthesis in the G1 phase. Flow cytometry was performed to analyze the apoptotic cells after FITC and PI staining. The results showed that high concentrations of ROB significantly increased the apoptotic rate of A549 and H1299 cells. And the apoptosis rate of A549 and H1299 cells increased most significantly at ROB concentration of 40 μM. (**Figures 12E–H**). It indicated that ROB could significantly stimulate apoptosis of lung cancer cells.

### 3.12 ROB induces autophagy of lung cancer cells

**Figure 13:**
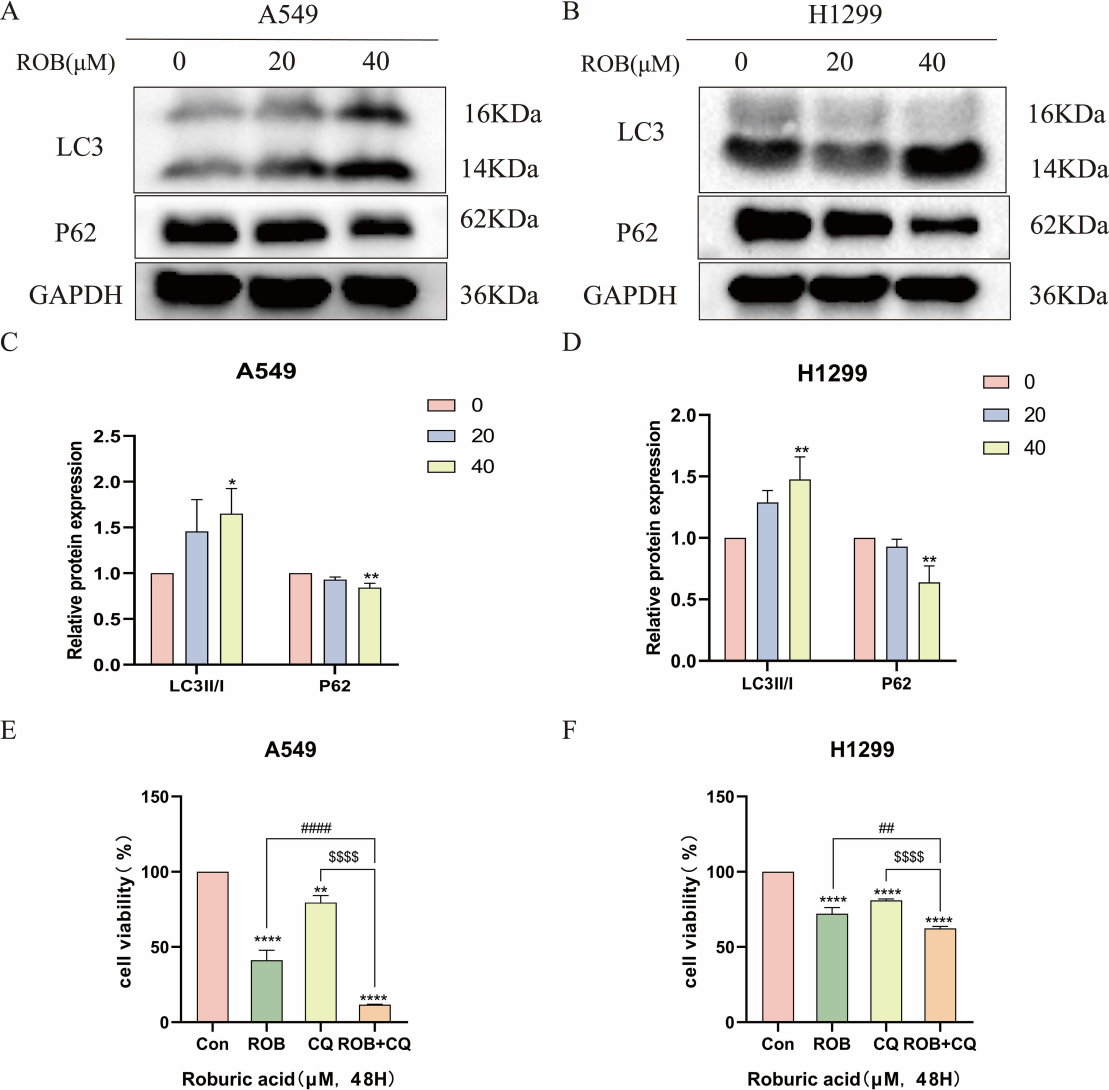
ROB induces autophagy in lung cancer cells. **(A–D)** The expression levels of LC3 and P62 proteins were detected by western blotting after 48 h of ROB action in lung cancer cells. *p < 0.05 and **p < 0.01 vs. control group. **(E, F)** After treating lung cancer cells with ROB, CQ, ROB and CQ respectively for 48 h, the cell viability of each group was detected by CCK8 assay. **p < 0.01 and ****p < 0.0001 vs. control group. ##p<0.01, ####p<0.0001 vs. ROB group; $$$$p< 0.0001 vs. CQ group.

We investigated whether ROB treatment induced autophagy in lung cancer cells. Autophagy was assessed by western blotting to detect changes in the autophagy markers LC3 and p62. After 48 h of ROB action, protein levels of LC3-II/I increased in a dose-dependent manner, while p62 decreased in the same manner (**Figures 13A–D**). Then, we assayed cell viability using the autophagy inhibitor Chloroquine (CQ, 10 μM) in combination with ROB and showed that ROB-induced inhibition of cell viability was significantly enhanced when ROB and CQ were combined (**Figures 13E, F**). These findings suggest that ROB induces autophagy in lung cancer cells and that autophagy plays a protective role in lung cancer cells.

### 3.13 ROB may inhibit metastasis and induce autophagy in lung cancer cells by regulating the PPARγ pathway

**Figure 14:**
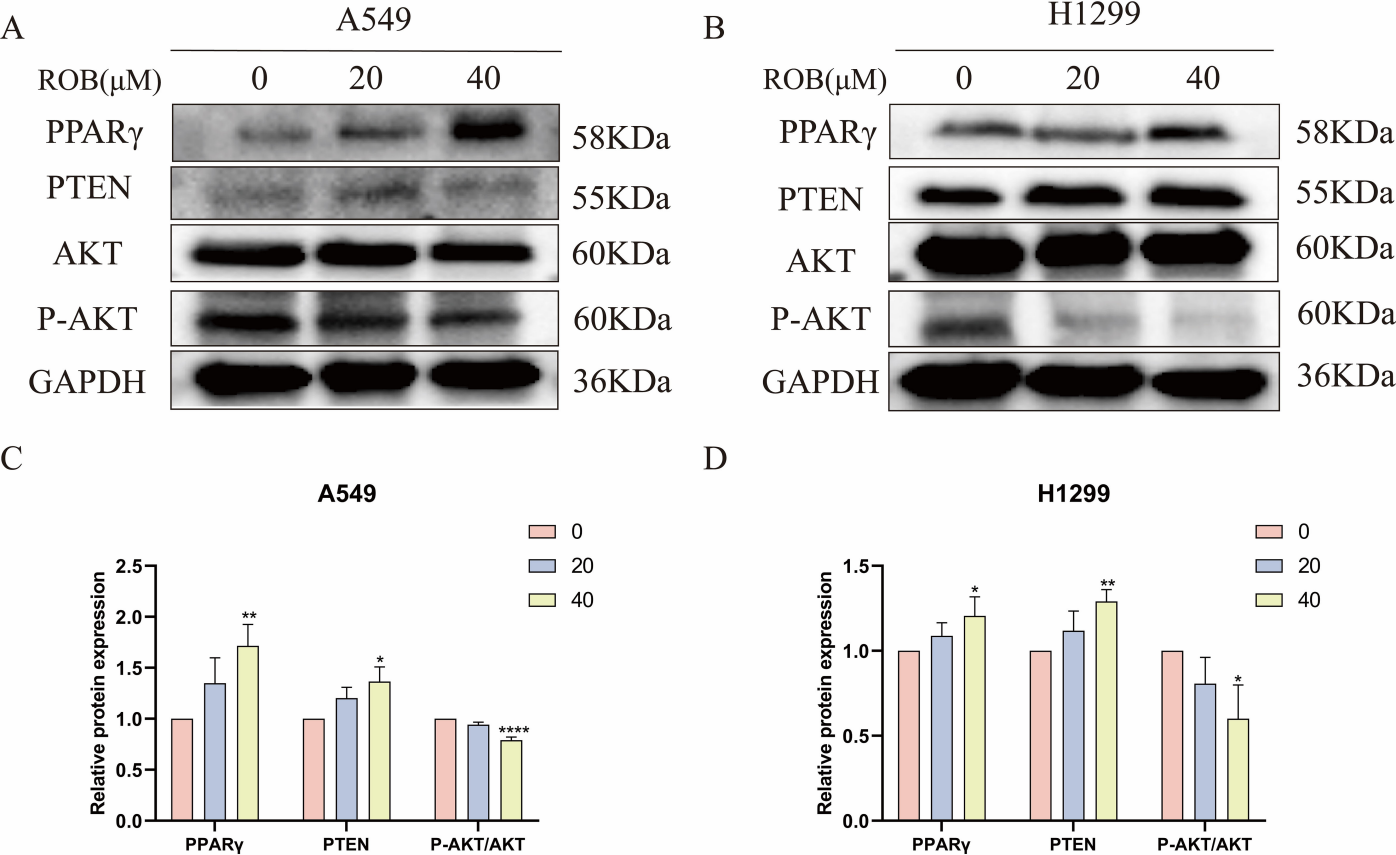
ROB inhibits lung cancer cell growth by regulating PPARγ pathway. **(A–D)** The expression levels of PPARγ, PTEN, AKT and P-AKT proteins were detected by western blotting after 48 h of ROB action in lung cancer cells. *p < 0.05, **p < 0.01 and ****p < 0.0001 vs. control group.

Based on the results of the previous network pharmacology KEGG analysis, it was hypothesized that ROB might treat lung cancer through the PPAR signaling pathway. To verify this possibility, we detected the expression of key proteins in the PPARγ pathway after ROB treatment for 48 h. The western blot results shown that the expression of PPARγ and PTEN was higher and the expression of p-Akt/Akt was lower in lung cancer cells treated with ROB compared with control cells (**Figure 14**). Therefore, we hypothesized that ROB may exert an inhibitory effect on lung cancer by activating the PPARγ/PTEN/Akt signaling pathway.

## 4 Discussion

Making for 18% of all cancer-related deaths, lung cancer is the primary cause of cancer-related mortality (19). Lung cancer is mostly treated with chemotherapy, but recent studies have shown that active compounds in traditional Chinese medicine have great potential in the treatment of lung cancer. It has been found that traditional Chinese medicine affects the proliferation and metastasis of lung cancer cells and improves the effectiveness of antitumor therapy by inducing or activating multiple tumor-related pathways. ROB is an active ingredient in *Gentiana macrophylla Pall.*, but its mechanism of action on lung cancer has not been fully clarified. Considering the better anti-inflammatory properties of ROB and the key role of the mediated NF-κB signaling pathway in the development and progression of colorectal cancer (20). However, to date, no studies have elucidated whether ROB have anti-lung cancer activity, nor have they identified their interaction targets and their potential mechanisms. Therefore, we utilized network pharmacology and molecular docking techniques to predict the targets and pathways of ROB in the treatment of lung cancer. We obtained 7 hub genes and PPAR signaling pathways for ROB action in lung cancer by network pharmacology analysis.

One of the leading causes of death in lung cancer patients is metastasis, making it one of the deadliest cancers in the world. Therefore targeted inhibition of metastasis may be a new direction in the treatment of lung cancer. In recent years, there has been increasing evidence that EMT is involved in the metastatic process (21). EMT is one of the important mechanisms of tumor metastasis; when extracellular factors stimulate tumor epithelial cells, they lose polarity, transform into mesenchymal cells, and exhibit enhanced migration and motility (22, 23). Typical EMT markers are E-cadherin, N-cadherin and vimentin. When tumor cells show down-regulation of E-cadherin expression, β-conjugated proteins on the cell membrane are transferred to the nucleus, leading to high expression of the mesenchymal cell markers N-cadherin and vimentin, which promotes tumor cell metastasis (24). The western blot assay was used for the detection of EMT marker proteins, and down-regulation of E-cadherin and up-regulation of N-cadherin and vimentin proteins were observed after ROB renting, suggesting that ROB inhibits the metastasis of lung cancer cells. In addition, after ROB acted on lung cancer cells, we further verified that ROB could inhibit the growth and metastasis of lung cancer cells using CCK8 assay, colony formation assay, wound healing and transwell assay.

In recent years, autophagy has emerged as a promising cancer therapeutic target, and inducing autophagy-associated cytotoxic death by blocking autophagic fluxes is increasingly recognized as a new cancer therapeutic strategy (25–27). During autophagy, LC3I binds to phosphatidylethanolamine (PE) and is converted to LC3II, which is then recruited to the autophagosome membrane (28). As a result, the amount of autophagy may be estimated using the LC3-II/I ratio (29). Enhanced autophagy or downstream degradation leads to accumulation of p62 (30). p62 eventually matures into autophagy *in vivo* and is degraded in autophagy (31, 32). The expression level of p62 was negatively correlated with autophagic activity and could be used as a complementary marker to monitor autophagy. Autophagy plays a pro-survival or pro-death role in drug-induced cytotoxicity in terms of cell type and/or drug-specific cytotoxicity (33), but it is unclear whether ROB action in lung cancer induces autophagy. We performed western blot assay to detect the expression of autophagy markers, and after ROB action with lung cancer cells, LC3-II/I was significantly elevated, while P62 was significantly reduced. A previous study showed that chemotherapeutic agents in combination with autophagy inhibitors such as chloroquine (CQ) inhibit the fusion of autophagosomes and lysosomes, thereby inhibiting the progression of autophagic flux and leading to cancer cell death (34, 35). Subsequently, we combined ROB and CQ with lung cancer cells and found that CQ enhanced the inhibitory effect of ROB. In summary, ROB can induce autophagy in lung cancer cells.

Next, we intend to reveal the underlying signaling pathways by which ROB functions in lung cancer. The PPAR signaling picture pathway was obtained through the analysis of network pharmacology. Numerous studies have demonstrated that the formation and progression of certain malignancies, including bladder cancer, astroglioma, hepatocellular carcinoma, and colorectal cancer, are typically associated with the aberrant regulation of the PPAR signaling pathway (36–39). Members of the nuclear hormone receptor superfamily include peroxisome proliferator-activated receptor (PPAR) (40). Of the three PPAR isoforms, PPARγ is more closely linked to cancer development. Therefore, we performed western blotting experiments, which showed that the protein levels of PPAR and PTEN were significantly reduced after ROB treatment, while the protein level of P-AKT/AKT was significantly elevated. These results suggest that ROB can inhibit lung cancer growth by activating the PPARγ/PTEN/Akt signaling pathway. Inhibitors or activators of the PPAR pathway can be added for further validation.

## 5 Conclusion

In summary, ROB inhibited the proliferation, migration, invasion and EMT of lung cancer cells and activated the PPARγ/PTEN/Akt signaling pathway, as well as induced autophagy in lung cancer cells. Therefore, in addition to the anti-inflammatory value of ROB, we found that it has potential anti-cancer value, providing new insights into the treatment of lung cancer and the search for new therapeutic approaches from TCM.

## Data Availability

The datasets presented in this study can be found in online repositories. The names of the repository/repositories and accession number(s) can be found in the article/**Supplementary Material**.
